# Presurgical therapy with axitinib for advanced renal cell carcinoma: a case report

**DOI:** 10.1186/1756-0500-6-484

**Published:** 2013-11-24

**Authors:** Takuya Koie, Chikara Ohyama, Akiko Okamoto, Hayato Yamamoto, Atsushi Imai, Shingo Hatakeyama, Takahiro Yoneyama, Yasuhiro Hashimoto

**Affiliations:** 1 Department of Urology, Hirosaki University Graduate School of Medicine, 5 Zaifucho, Hirosaki 036-8562, Japan

**Keywords:** Clear cell carcinoma, Axitinib, Presurgical therapy

## Abstract

**Background:**

Targeted therapy with tyrosine kinase inhibitors has been shown to reduce tumor volumes and prolong the survival of patients with metastatic renal cell carcinoma. Tyrosine kinase inhibitors, particularly sunitinib, have recently been used in neoadjuvant and presurgical settings. Axitinib is a promising second-line therapy option for advanced or metastatic renal cell carcinoma. Herein, we report a patient with advanced renal cell carcinoma who received presurgical treatment with axitinib.

**Case presentation:**

A 73-year-old man was transported by ambulance to a community hospital with chief complaints of high fever and a gait disorder. Computed tomography screening revealed a hypervascular tumor (size, 9 × 8.5 cm) in the lower pole of the left kidney. Upon admission to our hospital, his general condition was poor and his performance status was judged as 3, based on the Eastern Cooperative Oncology Group performance status criteria. After biopsy for the renal tumor, he received 5 mg of axitinib twice daily for 3 months. No serious adverse events were reported during this treatment. The tumor diameter shrank by 56%. Left radical nephrectomy was performed, and there were no intraoperative or postoperative complications. Pathological examination indicated a pT3aN0M0, Furman grade 3, clear cell renal cell carcinoma with necrosis, hyaline degeneration, and hemosiderosis. The patient was asymptomatic and disease-free at 1 year post-diagnosis.

**Conclusion:**

This case study demonstrate that presurgical therapy with axitinib is feasible and might have several potential advantages for patients with advanced renal cell carcinoma.

## Background

Targeted therapy with tyrosine kinase inhibitors (TKIs) has been shown to reduce primary or metastatic tumor volumes and prolong the survival of patients with metastatic renal cell carcinoma (RCC) [1,2]. TKIs, particularly sunitinib, have recently been used in neoadjuvant or presurgical settings to facilitate surgery by reducing tumor sizes [2,3]. The potential benefits of neoadjuvant or presurgical therapy include local tumor downstaging, elimination of micrometastatic disease, and diminution of postoperative metastatic progression.

However, targeted therapy with TKIs has elicited some concerns regarding complications. The commonly recorded grade 3/4 clinical toxicities associated with sunitinib were gastrointestinal disorders including diarrhea or anorexia, and hematotoxicity including neutropenia or thrombocytopenia [1]. Targeted therapy regimens should be less toxic to prevent any treatment-related delays between neoadjuvant therapy and surgery and to achieve better oncologic outcomes in a neoadjuvant setting.

Axitinib is a promising second-line therapy option for advanced or metastatic RCC [4]. In Japanese patients with metastatic RCC, adverse events with sunitinib was severer than the Caucasian population [5]. Additionally, this agent has shown anti-tumor activity and has an acceptable safety profile [6]. We designed a phase II single-arm trial to evaluate safety and effectiveness of presurgical treatment of advanced RCC with axitinib. This patient was enrolled this trial. Herein, we report a patient with advanced RCC who received presurgical treatment with axitinib. The patient was informed about the treatment protocol and provided written informed consent. The study protocol and informed consent documents were reviewed and approved by the Hirosaki University Institutional Review Board.

## Case presentation

**Figure 1:**
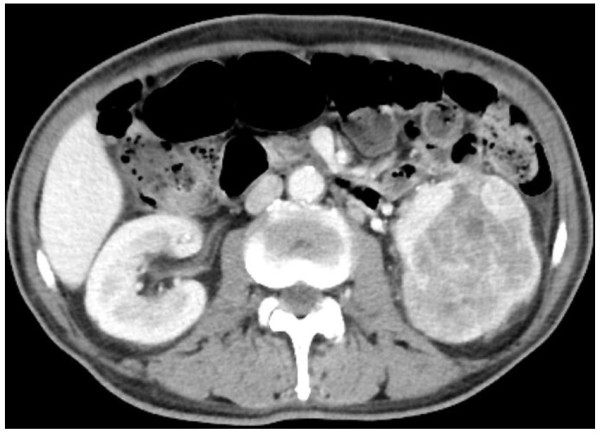
**Abdominal enhanced computed tomography (CT) before axitinib treatment.** (coronal section) Abdominal CT shows a hypervascular tumor (size, 9 × 8.5 cm) in the lower pole of the left kidney.

A 73-year-old man was transported by ambulance to a community hospital with chief complaints of high fever and a gait disorder. Laboratory evaluations revealed the following findings: hemoglobin, 7.0 g/dL (normal range, 13.5-17.5 g/dL); creatine phosphokinase, 572 IU/L (normal range, 60-270 IU/L); free blood sugar, 226 mg/dL (normal range, 70-109 mg/dL); and C-reactive protein, 14.0 mg/dL (normal range, <0.3 mg/dL). Computed tomography (CT) screening revealed a hypervascular tumor (size, 9 × 8.5 cm) in the lower pole of the left kidney (Figure 1). The tumor was clinically diagnosed as a left RCC with a classification of cT2aN0M0, according to the tumor-node-metastasis system [7]. The patient was transferred to our hospital for surgical treatment.

**Figure 2:**
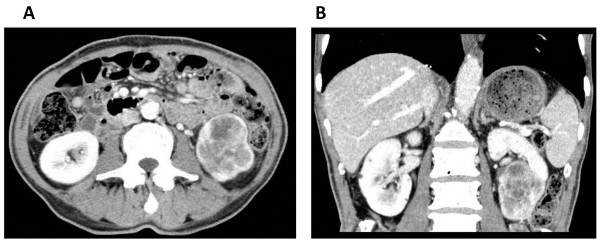
**Abdominal enhanced computed tomography (CT) after axitinib treatment.** (**A**, axial section; **B**, coronal section). Abdominal CT shows a hypervascular tumor (size, 4.5 × 2.5 cm) in the lower pole of the left kidney. The tumor diameter shrank by 56%, compared to that before axitinib treatment.

**Figure 3:**
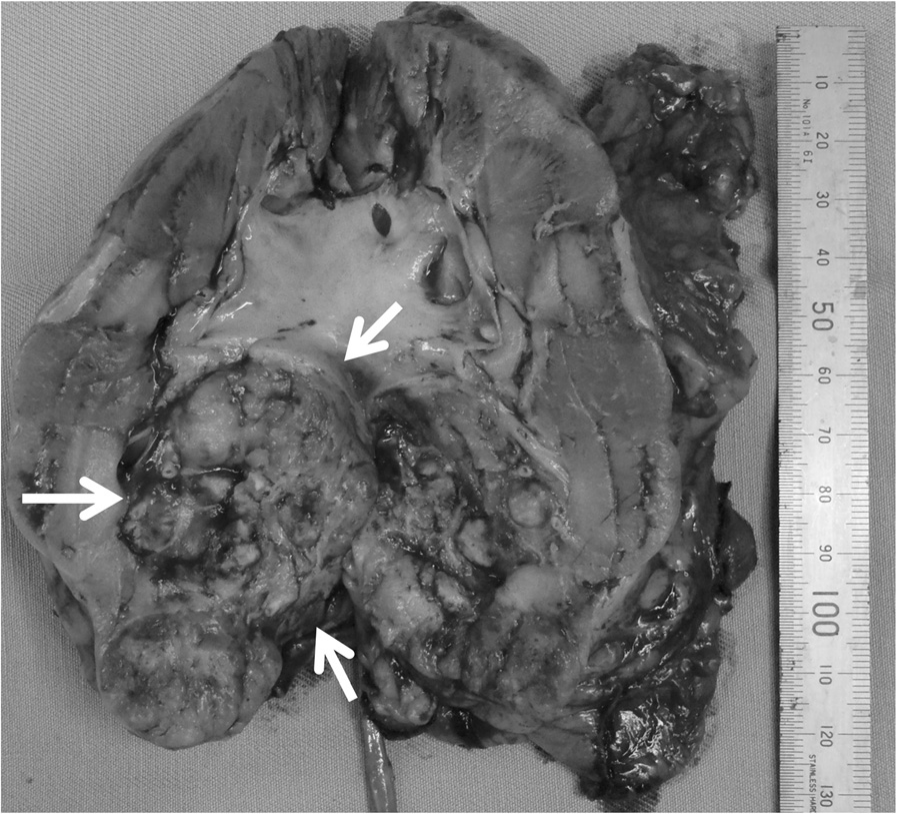
**Macroscopic findings.** Macroscopic examination reveals a solid tumor measuring 4 × 2.5 cm in size in the lower pole of the resected kidney (arrow).

**Figure 4:**
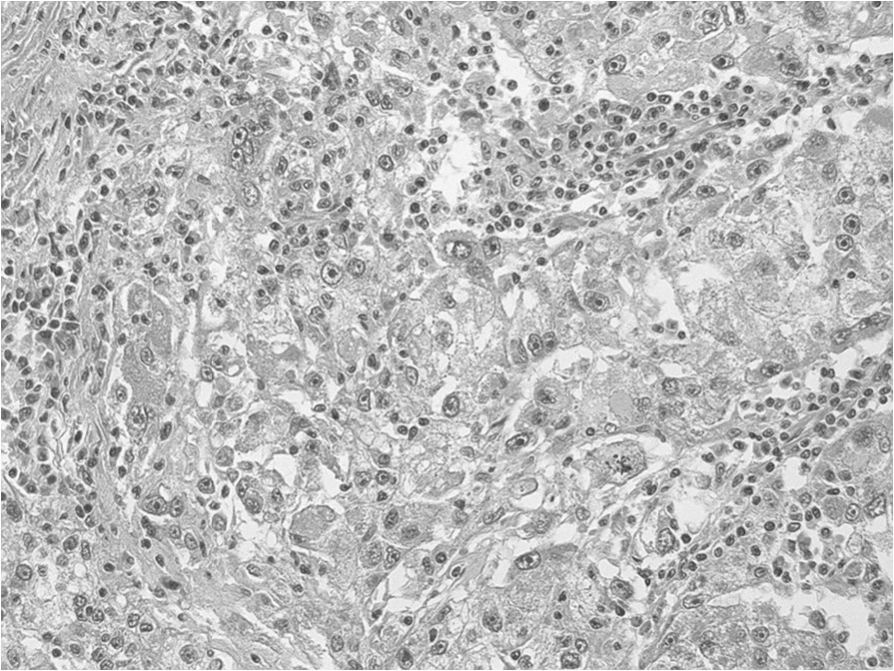
**Hematoxylin-eosin stained section.** The pathological findings indicate clear cell renal cell carcinoma with necrosis, hyaline degeneration, and hemosiderosis (magnification: ×200).

Upon admission to our hospital, the patient’s general condition was poor and his performance status (PS) was judged as 3, based on the Eastern Cooperative Oncology Group performance status criteria. Laboratory evaluations revealed the following findings: hemoglobin, 6.8 g/dL; adjusted calcium, 12.7 mg/dL (normal range, 8.7-10.3 g/dL); and alkaline phosphatase, 1670 U/L (normal range: 115-359 U/L). The patient underwent renal biopsy and was diagnosed with clear cell carcinoma of the left kidney. He received 5 mg of axitinib twice daily for 3 months, because we thought that immediate radical surgery was not safe because of his poor performance status and laboratory data paraneoplastic syndrome. Fortunately, no serious adverse events were reported during this treatment. The patient convalesced during the administration of axitinib, and all laboratory data were restored to within normal limits after axitinib treatment. An abdominal CT after the axitinib therapy showed that the tumor diameter shrank by 56% (Figure 2A, B). At that time, we were convinced that his renal tumor could be removed safely. Left radical nephrectomy was performed. The operation time was 95 min, and the estimated blood loss was 50 mL. No intraoperative or postoperative complications including wound healing delay or hemorrhage resulted from surgery. Macroscopic examination revealed a solid, yellowish-white tumor measuring 4 × 2.5 cm in size, with necrosis in the lower pole of the resected kidney (Figure 3). Pathological examination indicated pT3aN0M0, Furman grade 3, clear cell RCC with necrosis, hyaline degeneration, and hemosiderosis (Figure 4). Fifty percent of tumor tissue was necrosis, and it was central necrosis. The patient was asymptomatic and disease-free at 1 year post-diagnosis.

## Conclusions

To the best of our knowledge, this is the first reported case of presurgical axitinib therapy in a patient with RCC.

Targeted molecular therapies such as sunitinib have changed the management of advanced or metastatic RCC. TKIs have significantly improved progression-free survival, and sunitinib, in particular, achieved a median overall survival of >2 years in advanced RCC patients [8,9]. Therefore, neoadjuvant or presurgical treatments have been suggested as treatment options for patients with advanced or unresectable RCC. Several studies suggested that targeted therapies in the neoadjuvant setting were safe, well tolerated, and did not increase surgical morbidity or perioperative complications in selected patients [2,3,10]. However, these findings have been limited to small, uncontrolled, single-institution studies.

Potential disadvantages of presurgical or neoadjuvant targeted therapy include the lack of optimal surgical timing after systemic therapy and the risk of increased surgical morbidity [11]. Recent reports have cited complications such as poor wound healing and hemorrhage [12]. Chapin reported that presurgical targeted therapy remained a significant predictor of wound complications in a multivariate analysis (odds ratio, 4.14; *P* = 0.03) [13]. In a prospective study, the incidence of delayed superficial wound healing was higher in patients who received preoperative bevacizumab than in a historic matched cohort of 101 patients who underwent upfront surgery [14].

Axitinib is a potent and selective second-generation inhibitor of vascular endothelial growth factor receptor-1, 2, and 3 [15]. Axitinib has shown anti-tumor activity as a single agent with an acceptable safety profile in several solid tumors including previously treated metastatic RCC [4,6]. Adverse events observed in Japanese patients as well as in the overall axitinib-treated population, included diarrhea, hypertension, and fatigue [16]. Although axitinib was less effective for patients with poor PS [4], axitinib was generally well tolerated and had an acceptable safety profile in Japanese patients with metastatic RCC. Therefore, axitinib was administered for advanced RCC in this case.

In conclusion, this case study indicates that presurgical targeted therapy with axitinib might have several potential advantages for patients with advanced RCC. However, the indications and clinical benefits of neoadjuvant or presurgical axitinib therapy remain to be determined. It will thus be necessary to investigate many cases in a large clinical study.

## Consent

Written informed consent was obtained from the patient for the publication of this case report and any accompanying images. A copy of the written consent is available for review by the Editor-in-Chief of this journal.

## Abbreviations

CT: Computed tomography; RCC: Renal cell carcinoma; TKI: Tyrosine kinase inhibitor.

## Competing interests

The authors declare that they have no competing interests.

## Authors’ contributions

TK drafted the manuscript. OA, HY, TY, and AI performed clinical follow-up examinations and contributed to the manuscript. YH viewed the pathological specimens. CO, TK, and HS performed the surgeries. CO was responsible for the concept, design, data interpretation, and critical manuscript revision. All authors read and approved the final manuscript.
